# Supplementary material for: Transcriptome Analysis of Cabbage Near-Isogenic Lines Reveals the Involvement of the Plant Defensin Gene PDF1.2 in Fusarium Wilt Resistance
Source: Int J Mol Sci. 2025 Apr 16;26(8):3770. doi: 10.3390/ijms26083770 (PMC12028332; doi:10.3390/ijms26083770)
Supplement: Supplementary file 1 [file ijms-26-03770-s001.zip › ijms-3541536-supplementary.docx]

**Table S1.** RNASeq Map Statistics.

| **Sample ID** | **Clean_Reads No.** | **Total_Mapped** | **Uniquely_Mapped** |
| --- | --- | --- | --- |
| S-Foc-1 | 41427642 | 39289165 (94.84%) | 36196145 (92.13%) |
| S-Foc-2 | 40789702 | 38669803 (94.80%) | 35818524 (92.63%) |
| S-Foc-3 | 39572722 | 37190761 (93.98%) | 34756545 (93.45%) |
| S-MOCK1 | 36291182 | 34516583 (95.11%) | 32538035 (94.27%) |
| S-MOCK2 | 36646568 | 34731481 (94.77%) | 32966529 (94.92%) |
| S-MOCK3 | 45412050 | 43037317 (94.77%) | 40534166 (94.18%) |
| YR-Foc-1 | 36671228 | 34866450 (95.08%) | 33192777 (95.20%) |
| YR-Foc-2 | 38804600 | 36933933 (95.18%) | 34177699 (92.54%) |
| YR-Foc-3 | 45588484 | 43370583 (95.13%) | 41345899 (95.33%) |
| YR-MOCK1 | 36585610 | 34871856 (95.32%) | 33326371 (95.57%) |
| YR-MOCK2 | 35950636 | 33558278 (93.35%) | 32251659 (96.11%) |
| YR-MOCK3 | 37813296 | 35420701 (93.67%) | 34005845 (96.01%) |
| Total | 471553720 | 446456911（94.68%） | 421110194（94.32%） |

Note: Sample IDs are as follows: S-Foc corresponds to S01_20, S-MOCK corresponds to S01_20_MOCK, YR-Foc corresponds to YR01_20, YR-MOCK corresponds to YR01_20_MOCK. The terms used in the analysis are defined as follows: Clean Reads No.： the total number of paired-end reads in the clean data; Total Mapped: The total number of sequences successfully aligned to the reference genome, expressed as a percentage of clean reads (Total Mapped / Clean Reads); Uniquely Mapped: The total number of sequences that align to only one unique position in the reference genome, expressed as a percentage of total mapped sequences (Uniquely Mapped / Total Mapped).

**Table S2.** RNASeq Mapped Events.

| **Sample ID** | **Map_Events No.** | **Mapped_to_Gene** | **Mapped_to_Exon** |
| --- | --- | --- | --- |
| S-Foc-1 | 36196145 | 31647541 (87.43%) | 30024244 (94.87%) |
| S-Foc-2 | 35818524 | 31464045 (87.84%) | 30056377 (95.53%) |
| S-Foc-3 | 34756545 | 30588349 (88.01%) | 29419259 (96.18%) |
| S-MOCK1 | 32538035 | 28687166 (88.17%) | 27688594 (96.52%) |
| S-MOCK2 | 32966529 | 29113486 (88.31%) | 28228588 (96.96%) |
| S-MOCK3 | 40534166 | 35608540 (87.85%) | 34251491 (96.19%) |
| YR-Foc-1 | 33192777 | 29170058 (87.88%) | 28252501 (96.85%) |
| YR-Foc-2 | 34177699 | 29872909 (87.40%) | 28483488 (95.35%) |
| YR-Foc-3 | 41345899 | 36360382 (87.94%) | 35286445 (97.05%) |
| YR-MOCK1 | 33326371 | 29521839 (88.58%) | 28725675 (97.30%) |
| YR-MOCK2 | 32251659 | 28525008 (88.45%) | 27793935 (97.44%) |
| YR-MOCK3 | 34005845 | 29951761 (88.08%) | 29073640 (97.07%) |
| Total | 421110194 | 370511084（87.98%） | 357284237（96.43%） |

Note: Sample IDs are as follows: S-Foc corresponds to S01_20, S-MOCK corresponds to S01_20_MOCK, YR-Foc corresponds to YR01_20, YR-MOCK corresponds to YR01_20_MOCK. The terms used in the analysis are defined as follows: Map Events No.: the total number of mapping events that have occurred; Mapped to Gene: The total number of reads mapped to gene regions, expressed as a percentage of total mapping events (Mapped to Gene / Map Events); Mapped to Exon: The total number of reads mapped to exon regions, expressed as a percentage of reads mapped to gene regions (Mapped to Exon / Mapped to Gene).


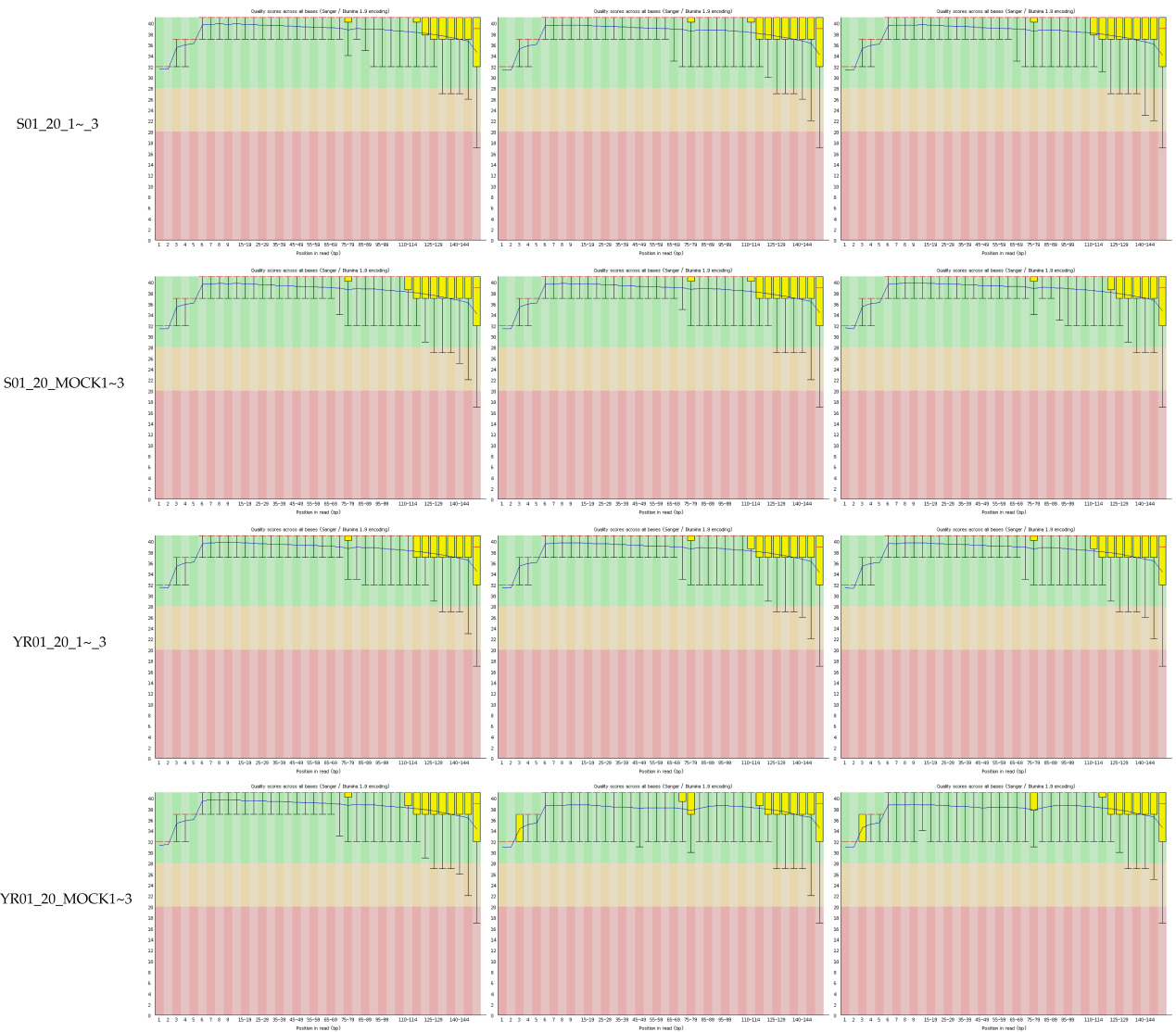


**Figure S1.** Single base quality distribution chart. Note: The horizontal axis represents the base position in Reads (5'→3'), and the vertical axis represents the Q value of the corresponding base site. The red line indicates the median, the blue line indicates the average, the yellow area represents the 25%-75% interval (divided by quartiles), and the whiskers represent the 10%-90% interval. Repeat1 to Repeat3 represent the three biological replicates of each group.


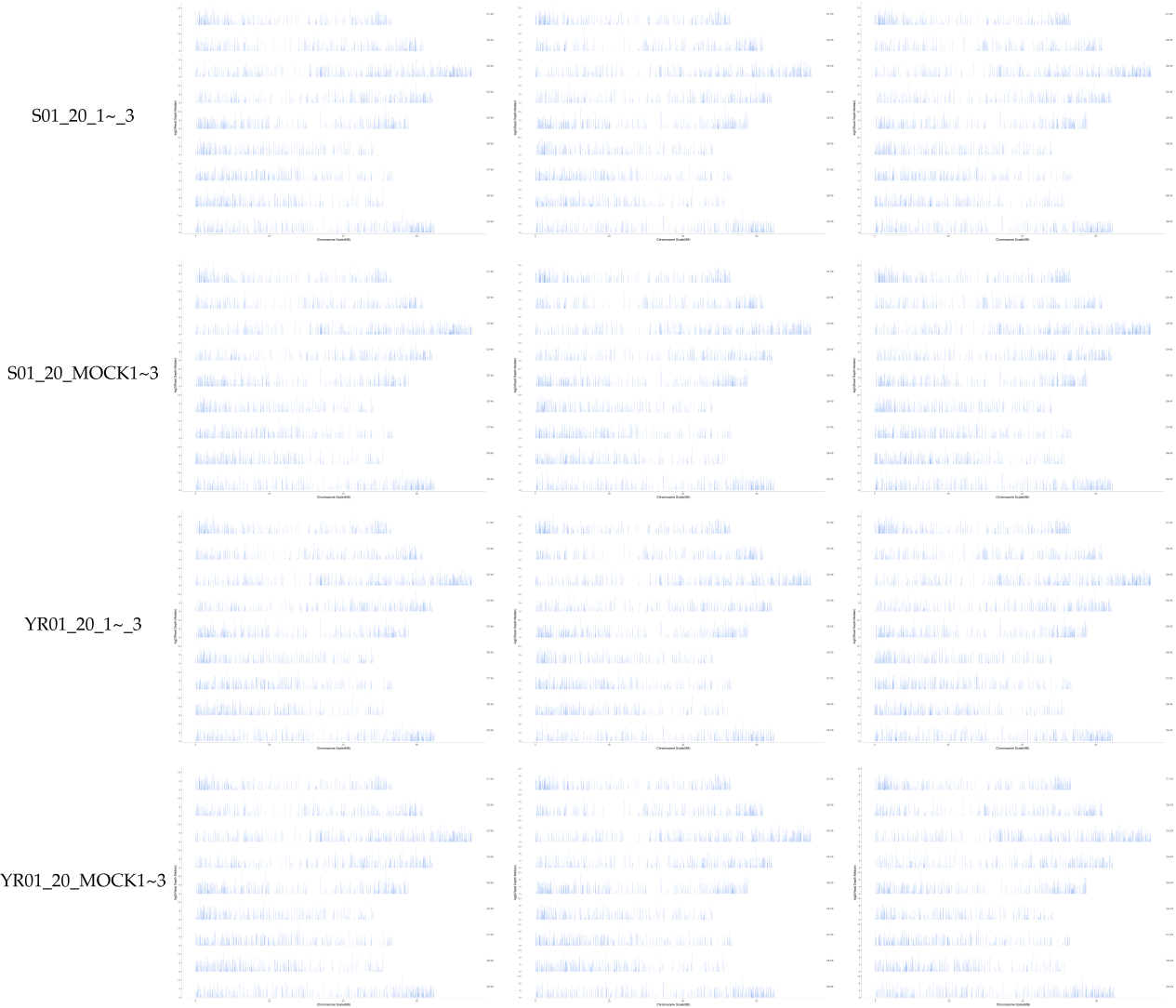


**Figure S2.** Reads distribution map on chromosomes.


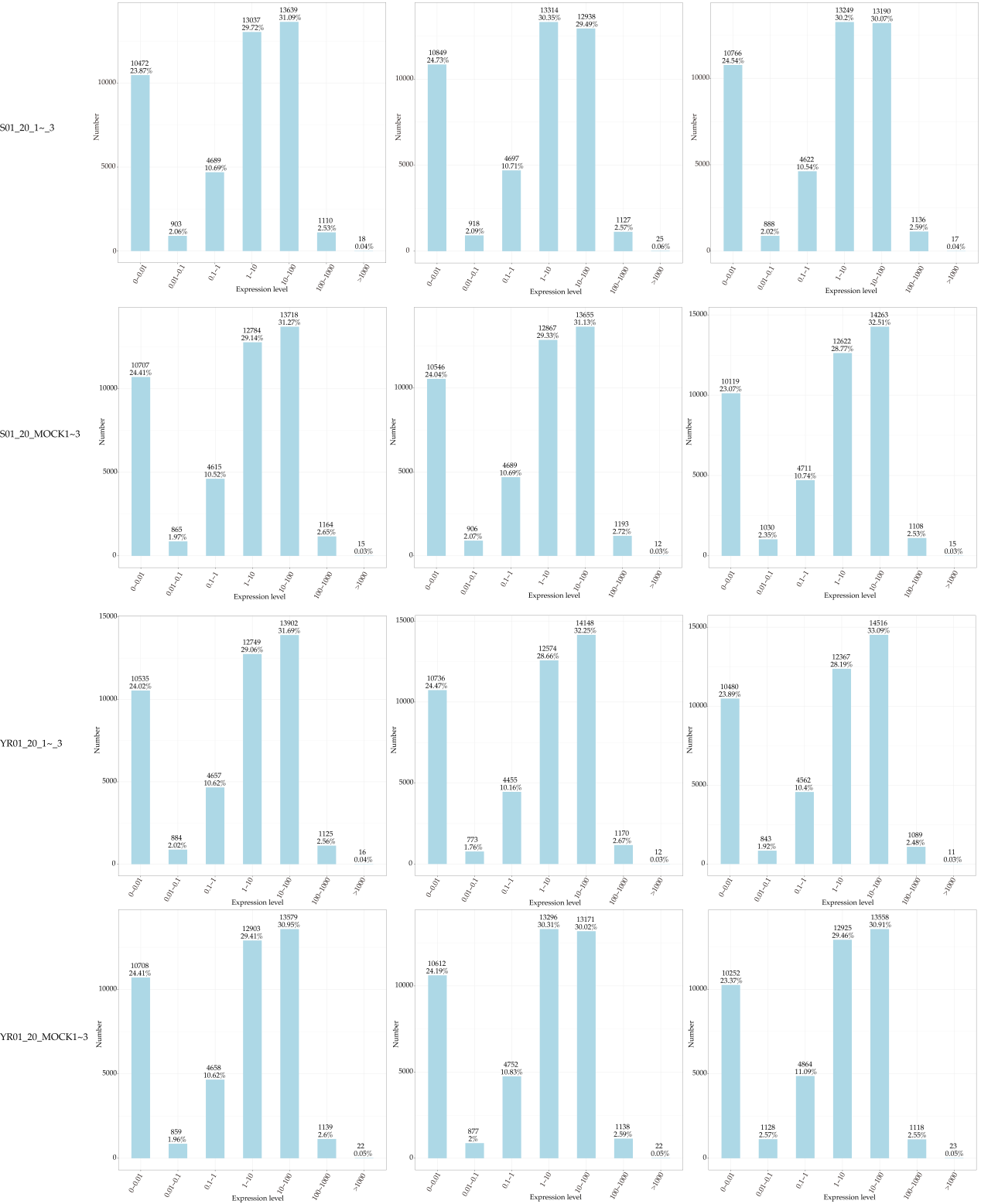


**Figure S3.** Distribution of Gene Expression Levels. Quantitative stratification of gene expression patterns across experimental samples. Expression levels derived from RNA-seq data (Figure 1A) were logarithmically stratified into discrete intervals. Histogram bins represent absolute gene counts per expression level range (log2-transformed FPKM values) aggregated across all biological replicates. Note: The x-axis denotes the intervals of expression levels, while the y-axis indicates the count of genes falling within per expression level interval.


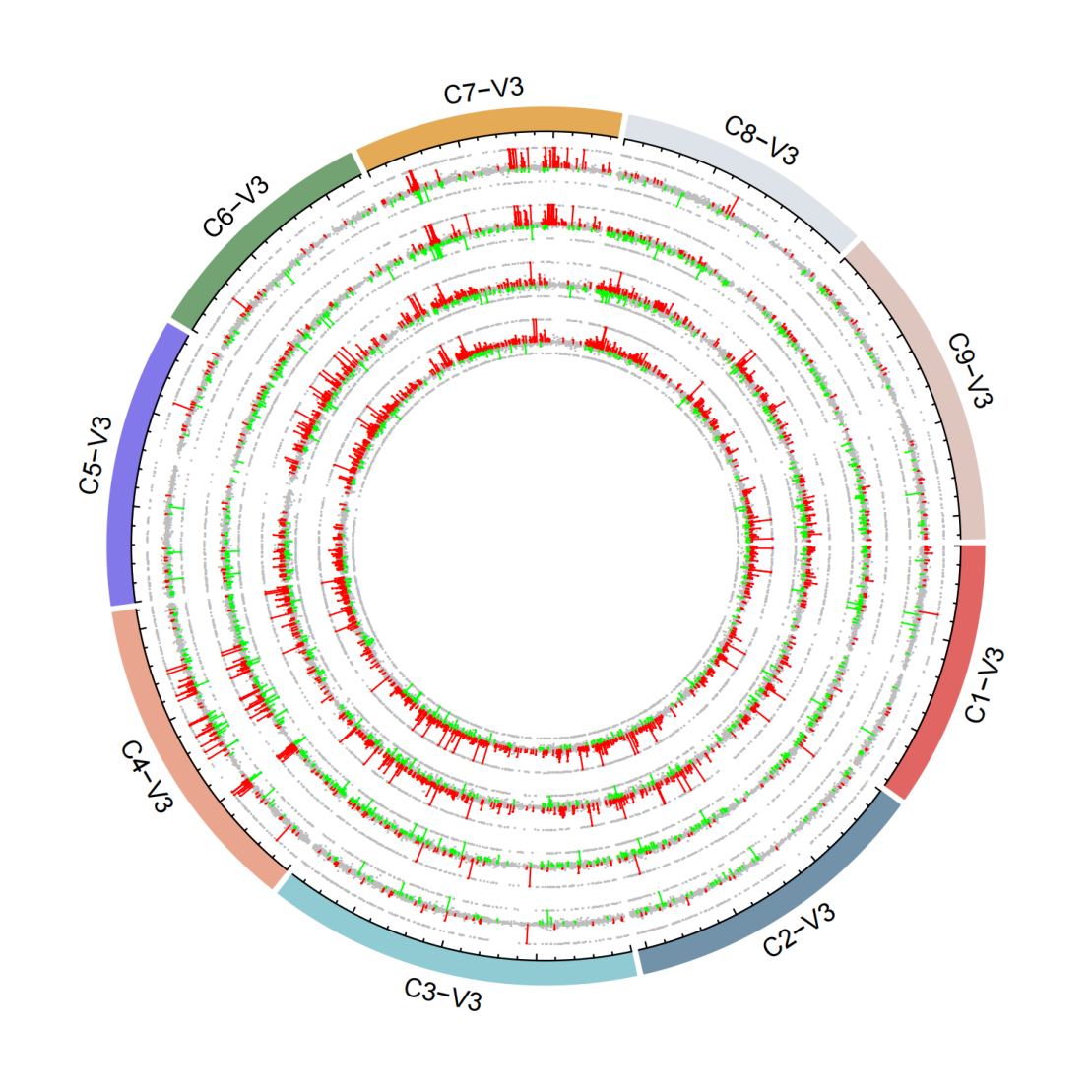


**Figure S4.** Genome Circle Plot. The outermost circle represents the chromosome bands. Moving inward, the subsequent circles depict the differential expression analysis results for the following comparisons: S01_20_vs_YR01_20, S01_20_MOCK_vs_YR01_20_MOCK, S01_20_MOCK_vs_S01_20, and YR01_20_MOCK_vs_YR01_20. Red and green bars represent the log2FoldChange values of up-regulated and down-regulated genes, respectively, while gray dots indicate the log2FoldChange values of genes with non-significant differential expression.


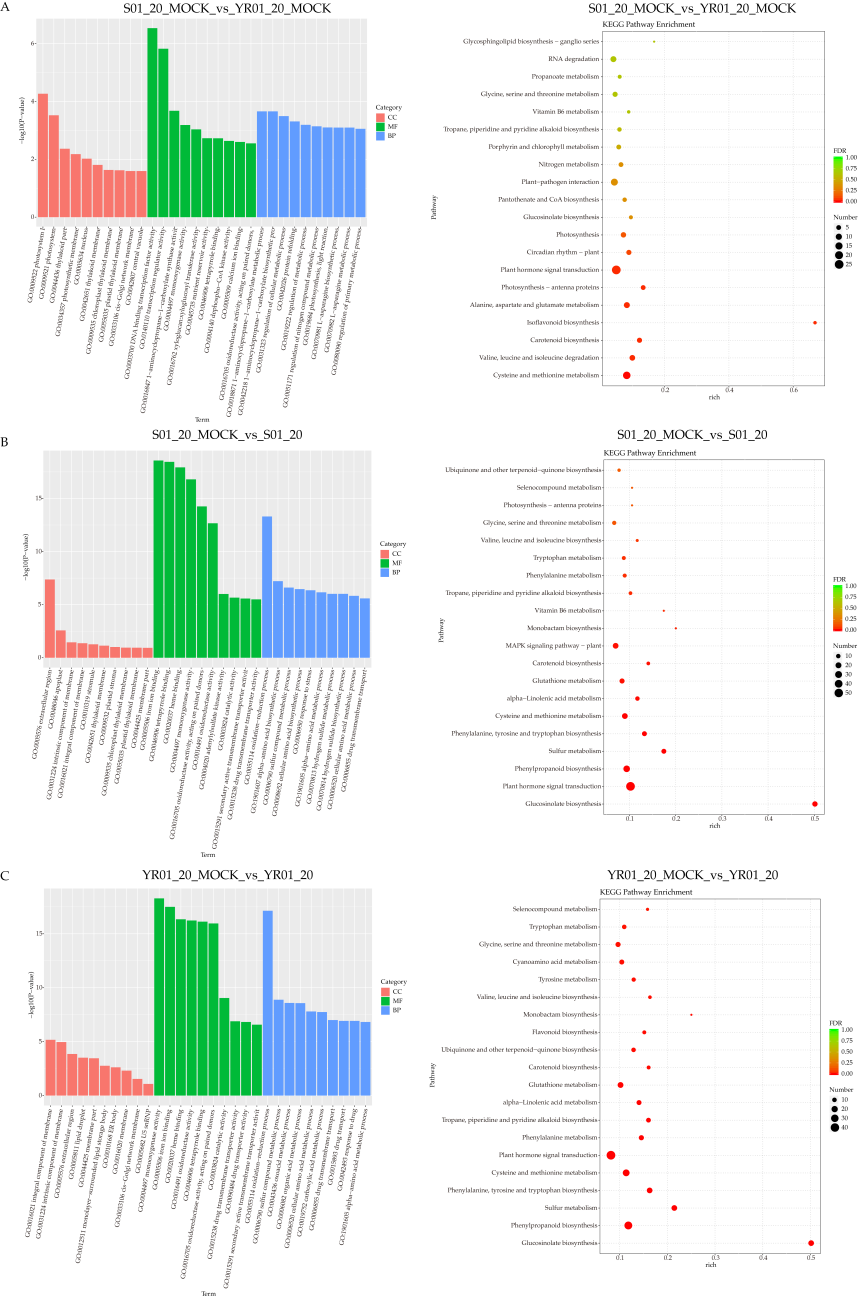


**Figure S5.** Functional Enrichment Analysis of Differentially Expressed Genes. (A, B, C) GO annotation classification (left panel) and KEGG enrichment bubble plot (right panel) of differentially expressed genes in S01_20_MOCK_vs_YR01_20_MOCK, S01_20_MOCK_vs_S01_20, and YR01_20_MOCK_vs_YR01_20. Bar plot showing the top 10 significantly enriched terms in the three categories of cellular component, biological processes, and molecular function for DEGs. Bar height represents the enrichment factor [(Number of DEGs in term)/(Number of background genes in term)]. Terms with FDR-adjusted p-value < 0.05 (Benjamini-Hochberg method) were considered significant, with level 2 annotations highlighting broad functional classifications. The bubble plot visualizes the top 20 significantly enriched KEGG pathways. The x-axis represents the enrichment factor (calculated as [DEGs in pathway]/[background genes in pathway]), while the y-axis displays pathway nomenclature sorted by descending enrichment factor. Bubble diameter correlates with the number of differentially expressed genes (DEGs) annotated per pathway. A color gradient from red to yellow reflects ascending FDR thresholds (crimson: FDR < 0.01; amber: FDR < 0.05; pale yellow: FDR ≥ 0.05), with color intensity scaled proportionally to -log10(FDR) values. Gray dashed lines demarcate the conventional significance threshold (FDR = 0.05, Benjamini-Hochberg adjusted).
